# The association of thyroid stimulating hormone and body fat in adults

**DOI:** 10.1371/journal.pone.0314704

**Published:** 2024-12-03

**Authors:** Qin Sun, Yixuan He, Liang Yang

**Affiliations:** 1 Yulin Hospital, The First Affiliated Hospital of Xi’an Jiaotong University, Xi’an, Shaanxi, China; 2 Chinese Academy of Medical Sciences & Peking Union Medical College, Peking Union Medical College Hospital, Beijing, Beijing, China; Tribhuvan University Institute of Medicine, NEPAL

## Abstract

**Background:**

Thyroid stimulating hormone (TSH) has been proven to be closely associated with metabolic abnormalities, including obesity. The objective of this investigation was to scrutinize the intricate association between TSH concentration and obesity, within the adult population. The study focused on a comprehensive examination of the relationship, delving into specific adiposity parameters such as total percent fat (TPF), android percent fat (APF), and gynoid percent fat (GPF).

**Methods:**

This study included 809 participants aged 20 and above with normal TSH values from the National Health and Nutrition Examination Survey (NHANES) 2007–2012. Multivariable linear regression models examined the associations of TSH concentration with TPF, APF, and GPF. Subgroup analyses, stratified by sex, were performed using multivariable linear regression. Fitted smoothing curves and generalized additive models addressed non-linear relationships between TSH and TPF, APF, and GPF.

**Results:**

In fully adjusted models, a significant positive association was observed between TPF and TSH (β = 0.01, 95% CI: 0.00–0.02, p<0.05), while no such association was evident in APF and GPF. Upon sex stratification, females exhibited significant positive correlations between TSH and TPF, APF, and GPF (all p < 0.001), contrasting with males where no such correlations were found. Notably, a non-linear association was identified in males, specifically a U-shaped curve (inflection point: 32.6%) for TSH and APF.

**Conclusion:**

The study unveiled a statistically significant positive association between TSH and TPF in adults. Upon sex stratification, similar statistically significant relationships were observed between TSH and adiposity (TPF, APF, GPF) in females, while males exhibited a U-shaped non-linear relationship between TSH and APF.

## Introduction

Obesity has emerged as a prominent global health concern, characterized by the abnormal or excessive accumulation of fat, posing significant health risks. The prevalence of obesity has surged nearly threefold since 1975 on a global scale, with projections indicating a continued rise, anticipating one in five adults to be obese by 2025 [1]. Alarming statistics also highlight the substantial burden on children, with 39 million under the age of 5 being overweight or obese in 2020 [2]. The implications of obesity extend across various organs, leading to complications such as hypertension, diabetes, and heart disease. Currently, the assessment of obesity commonly relies on body mass index (BMI) due to its simplicity and convenience. However, emerging research suggests that fat distribution serves as a more precise and accurate predictor of numerous diseases, including cardiovascular disease, compared to BMI [3]. Consequently, there is a growing interest in dual-energy x-ray absorptiometry (DXA) as a technique capable of accurately measuring regional fat content. Researchers have extensively explored the intricate relationships between body composition, measured precisely by DXA, and diseases like diabetes, and hyperlipidemia [4, 5].

Thyroid-stimulating hormone (TSH), secreted by the pituitary gland, undergoes regulation through thyrotropin-releasing hormone (TRH) from the hypothalamus and thyroid hormones (FT3 and FT4) from the thyroid. Deviations in TSH levels may signify thyroid dysfunction, with even minor disturbances holding significant implications for various clinical endpoints, including bone mineral density, depression, metabolic syndrome, and cardiovascular disease [6]. The determination of the upper limit for normal TSH values remains a subject of controversy, as several factors can influence TSH levels. Studies have indicated an age-related increase in TSH [7], as well as associations with BMI [8]. Sex differences further contribute to variations in TSH concentration, with females generally exhibiting higher levels than males. Additionally, females are more predisposed to developing thyroid-related diseases, potentially attributed to differences in immune function, as evidenced by their higher likelihood of possessing TPO antibodies compared to males [9].

Hence, exploring the associations between TSH concentrations and body fat is essential, especially as previous studies on this topic have often been limited by small sample sizes and a reliance on BMI, which may not fully capture regional adiposity. The use of DXA in our study offers a more precise assessment of body fat distribution, addressing these limitations by providing detailed measures of regional adiposity. Moreover, while some research has acknowledged the role of sex in the TSH-adiposity relationship, findings have been inconsistent or inadequately stratified by sex. By leveraging the extensive and representative NHANES dataset, our analysis not only examines these associations comprehensively but also stratifies results by sex to reveal distinct trends in TSH and body fat distribution across genders.

## Materials and methods

### Study population

**Fig 1:**
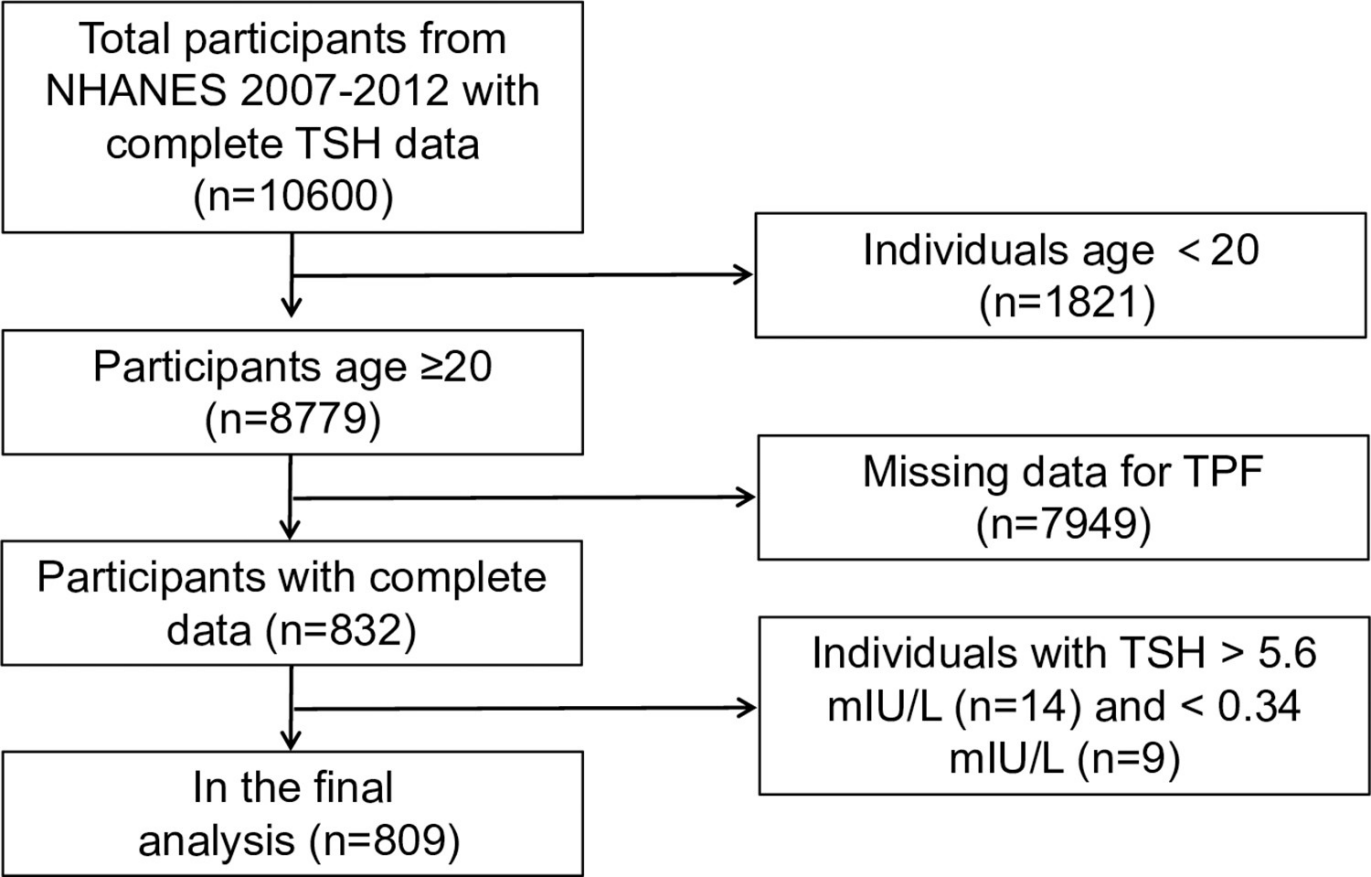
Flow chart of sample selection from the NHANES 2007–2012.

Data of participants aged 18–59 years with complete TSH data were retrieved from the 2007–2012 National Health and Nutrition Examination Survey (NHANES), a comprehensive program combining interviews and physical examinations to evaluate the health and nutritional status of individuals in the United States. Initially comprising 10,600 participants, the final analysis included 8,049 individuals after excluding 1,821 aged below 20 years, 7,949 participants without total percent fat (TPF) data, and 23 individuals with abnormal TSH data (14 with TSH above 5.6 mlU/L and 9 with TSH below 0.34 mlU/L). The National Center for Health Statistics Research Ethics Review Board thoroughly reviewed and approved NHANES, with all participants voluntarily signing the informed consent form **(Fig 1)**.

### Variables

The exposure variable, adiposity, encompassing TPF, APF, and GPF, was quantified using Hologic Discovery model A densitometers (Hologic, Inc., Bedford, Massachusetts) [10]. The measurement process employed software version Apex 3.2 and was conducted by proficient radiology technologists with certification and training. TPF was computed as the ratio of total fat mass to the combined mass of fat and lean tissue, multiplied by 100 to yield a percentage. The delineation of android and gynoid (A/G) regions was facilitated by the Hologic APEX software during scan analysis. The android area was defined as the lower trunk region between the pelvic horizontal cut line and a line automatically positioned above the pelvic line. The upper gynoid line was set at 1.5 times the height of the android region below the pelvic line, while the lower gynoid line was positioned to maintain a distance between the two lines twice the height of the android region. All line placements were automated functions of the Hologic software.

The outcome variable, TSH, was ascertained from blood specimens utilizing the Access HYPER-sensitive human thyroid-stimulating hormone (hTSH) assay. The assay involves binding TSH in the serum to monoclonal antibodies on a solid phase, followed by a chemiluminescent reaction that produces light proportional to the TSH concentration, which is then measured with a luminometer. This method offers a detection limit of 0.01 μIU/mL and maintains precision with less than 10% coefficient of variation, ensuring reliable quantification across a broad range of TSH concentrations. Detail description is available on the NHANES website (http://www.cdc.gov/nchs/nhanes/). Other variables included in our analysis encompassed age, sex, race, BMI, ratio of family income to poverty, marital status, vigorous work activity, smoking status (defined as having smoked at least 100 cigarettes in life), hypertension (self-reported physician-diagnosed hypertension), diabetes (self-reported diagnosed diabetes or prediabetes), hyperlipidemia (self-reported high blood lipid as diagnosed by a doctor). Arm circumference and waist circumference, obtained during the NHANES examination, were also considered.

Several metabolic indicators were available through the NHANES laboratory component, including HDL (measured by the polyethylene glycol-modified enzyme method using the Cobas 6000 Chemistry Analyzer), TC (measured by the Trinder reaction using the Roche Modular P Chemistry Analyzer), TG (measured by the enzymatic triglyceride assay on the Roche Modular P Chemistry Analyzer), LDL (calculated as [TC]—[HDL]—[TG/5]), creatinine (measured by the Jaffe rate method using the Beckman UniCel® DxC800 Synchron), blood urea nitrogen (measured by the urease conductivity method on the Beckman UniCel® DxC800 Synchron), and serum uric acid (measured by the uricase oxidation method using the Beckman UniCel® DxC800 Synchron).

### Statistical analyses

Baseline characteristics of the participants in this study group were presented as means ± SDs (standard deviation) for continuous variables and percentages for categorical variables. TSH concentrations were categorized into low-, middle-, and high-normal groups, following the methodology outlined by Kosuke’s study [11]. All statistical analyses were conducted using the R package (http://www.Rproject.org) and Empower Stats (http://www.empowerstats.com), with a significance threshold set at p < 0.05. Multivariable linear regression models were employed to assess the associations of TSH with TPF, APF, and GPF. Three models were constructed: unadjusted model 1, minimally adjusted model 2 (controlling for age, sex, and race), and fully adjusted model 3 (controlling for age, sex, race, hypertension, diabetes, hyperlipidemia, marital status, smoking status, and vigorous work activity). Subgroup analyses stratified by sex were also conducted.

## Result

**Table 1** presents the baseline characteristics of participants in this study for each variable. Notably, participants in the higher TSH group exhibited higher values for BMI, arm circumference, waist circumference, TPF and APF. This trend was less pronounced in GPF. Intergroup comparisons further revealed that metabolic indicators, including triglycerides and total cholesterol, increased with elevated TSH levels, while High-Density Lipoprotein (HDL) showed a negative correlation. Additionally, participants with higher TSH concentrations had a higher prevalence of hypertension and diabetes, along with a lower prevalence of smoking. In contrast, the middle-normal TSH concentration group exhibited the highest prevalence of hyperlipidemia and vigorous work activity.

**Table 1:** Baseline characteristics of 809 patients according to TSH concentrations.

	TSH Concentration (mIU/L)		
Characteristic	Low-Normal	Middle-Normal	High-Normal
Participants (No.)	283	317	209
TSH range (mIU/L)	0.34–1.19	1.20–1.95	1.96–5.60
TSH concentration (mIU/L)	0.89 ± 0.22	1.55 ± 0.22	2.79 ± 0.81
Total percent fat (%)	31.88 ± 7.60	31.83 ± 7.86	32.96 ± 8.82
Android percent fat (%)	33.69 ± 8.64	34.28 ± 8.22	35.04 ± 9.41
Gynoid percent fat (%)	35.00 ± 8.21	34.00 ± 8.38	35.40 ± 9.05
Age (years)	38.37 ± 11.71	40.21 ± 11.92	39.89 ± 12.11
Ratio of family income to poverty	3.04 ± 1.74	2.98 ± 1.70	2.93 ± 1.70
BMI (kg/m^2^)	27.57 ± 5.72	28.65 ± 6.21	29.24 ± 7.14
Arm circumference (cm)	32.72 ± 4.85	33.66 ± 4.65	34.04 ± 4.92
Waist circumference (cm)	95.33 ± 15.31	97.64 ± 15.92	98.83 ± 16.90
HDL Cholesterol (mmol/L)	1.40 ± 0.46	1.32 ± 0.34	1.32 ± 0.32
LDL Cholesterol (mmol/L)	2.95 ± 0.85	3.21 ± 0.96	3.11 ± 0.94
Triglycerides (mmol/L)	1.56 ± 1.21	1.69 ± 1.26	1.95 ± 2.19
Total Cholesterol (mmol/L)	4.94 ± 1.06	5.12 ± 1.03	5.17 ± 1.09
Creatinine (μmol/L)	75.16 ± 32.92	76.41 ± 21.17	75.74 ± 16.41
Blood urea nitrogen (mmol/L)	4.06 ± 1.35	4.46 ± 1.56	4.41 ± 1.53
Serum uric acid (μmol/L)	305.28 ± 76.75	326.47 ± 90.13	323.49 ± 75.72
Sex			
Males	50.33	54.30	52.56
Females	49.67	45.70	47.44
Race (%)			
Hispanic	15.78	14.18	14.00
Non-Hispanic White	59.83	67.95	73.38
Non-Hispanic Black	15.50	9.91	6.74
Others	8.89	7.96	5.88
Marital status			
Married or with a partner	58.38	62.92	57.70
Others	41.62	37.08	42.30
Hypertension (%)			
Yes	18.90	22.95	23.76
No	81.10	77.05	76.24
Hyperlipidemia (%)			
Yes	22.11	35.74	27.59
No	77.89	64.26	72.41
Diabetes (%)			
Yes	9.27	10.16	10.51
No	90.73	89.84	89.49
Smoking status (%)			
Yes	45.31	37.30	36.58
No	54.69	62.70	63.42
Vigorous work activity (%)			
Yes	22.49	29.35	20.71
No	77.51	70.65	79.29

**Notes:** Mean ± SD for continuous variables and P value was calculated by weighted linear regression model. % for categorical variables and P value was calculated by weighted chi-square test.

In both model 1, without covariate adjustment, and model 2, adjusted for age, sex, and race, TPF demonstrated a positive association with TSH (model 1: β = 0.01, 95% CI: 0.00–0.02; model 2: β = 0.01, 95% CI: 0.00–0.02). This positive association persisted in the fully adjusted model 3 (model 3: β = 0.01, 95% CI: 0.00–0.02). However, APF and GPF exhibited no significant relationship with TSH in these models.

Notably, intriguing findings emerged upon stratification by sex. Among males, no statistically significant relationships were observed between TPF, APF, GPF, and TSH in all three models. Conversely, in females, statistically significant positive correlations were evident in all models (**TPF:** model 1: β = 0.03, 95% CI: 0.02–0.04; model 2: β = 0.03, 95% CI: 0.02–0.05; model 3: β = 0.03, 95% CI: 0.02–0.05; **APF:** model 1: β = 0.02, 95% CI: 0.01–0.03; model 2: β = 0.02, 95% CI: 0.01–0.03; model 3: β = 0.02, 95% CI: 0.01–0.03; **GPF:** model 1: β = 0.03, 95% CI: 0.01–0.04; model 2: β = 0.03, 95% CI: 0.01–0.04; model 3: β = 0.03, 95% CI: 0.01–0.05). **Table 2** comprehensively presents all the mentioned results.

**Table 2:** The associations between body fat composition and TSH (mIU/L).

	Model 1	Model 2	Model 3
	β (95% CI) P value	β (95% CI) P value	β (95% CI) P value
**Total**			
TPF (%)	0.01 (0.00, 0.02) 0.0151	0.01 (0.00, 0.02) 0.0296	0.01 (0.00, 0.02) 0.0338
APF (%)	0.01 (0.00, 0.02) 0.0435	0.01 (-0.00, 0.02) 0.0773	0.01 (-0.00, 0.01) 0.1010
GPF (%)	0.01 (-0.00, 0.02) 0.1873	0.01 (-0.01, 0.02) 0.2649	0.01 (-0.00, 0.02) 0.2194
**Sex**			
** Males**			
TPF (%)	-0.00 (-0.02, 0.01) 0.6820	-0.01 (-0.02, 0.01) 0.3486	-0.01 (-0.02, 0.01) 0.3051
APF (%)	-0.00 (-0.01, 0.01) 0.5578	-0.01 (-0.02, 0.01) 0.2750	-0.01 (-0.02, 0.00) 0.2066
GPF (%)	-0.01 (-0.03, 0.01) 0.2033	-0.01 (-0.03, 0.00) 0.0908	-0.01 (-0.03, 0.00) 0.1111
** Females**			
TPF (%)	0.03 (0.02, 0.04) <0.0001	0.03 (0.02, 0.05) <0.0001	0.03 (0.02, 0.05) <0.0001
APF (%)	0.02 (0.01, 0.03) 0.0002	0.02 (0.01, 0.03) 0.0002	0.02 (0.01, 0.03) 0.0003
GPF (%)	0.03 (0.01, 0.04) 0.0006	0.03 (0.01, 0.04) 0.0005	0.03 (0.01, 0.05) 0.0002

**Notes:** No covariate was adjusted in Model 1. Model 2 indicates that analysis was adjusted for age, sex, and race. Model 3 indicates model 2 adjustment plus the adjustment for hypertension, diabetes, hyperlipidemia, marital status, smoking status, vigorous work activity. Sex was not adjusted in the sex-stratified subgroup analyses.

**Fig 2:**
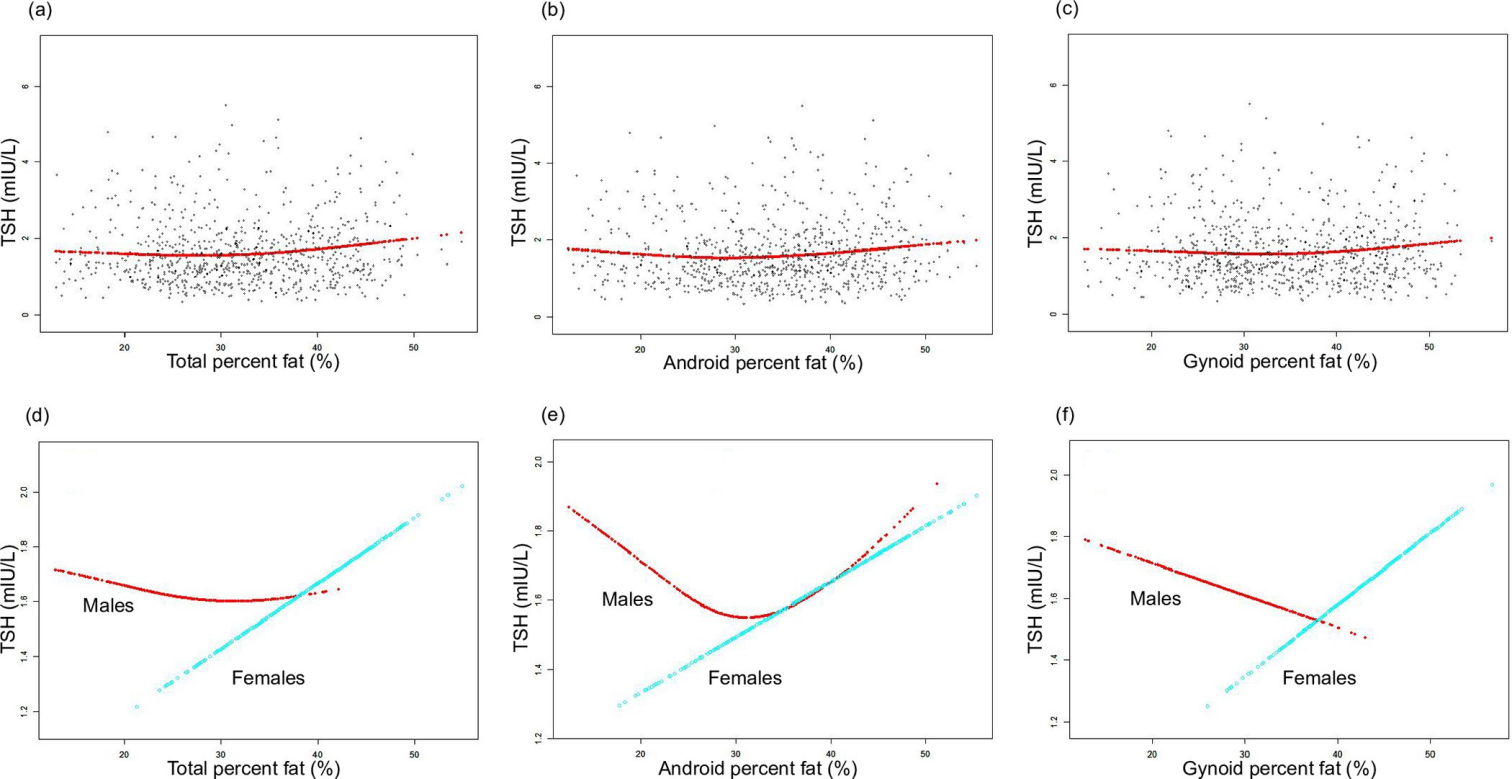
The associations between body fat composition and TSH (mIU/L). (a)(b) (c) Each black point represents a sample. (d) (e) (f) Associations of TPF, APF, and GPF with TSH stratified by sex. Age, race, hypertension, diabetes, hyperlipidemia, marital status, smoking status and vigorous work activity were adjusted.

The non-linear relationships between TPF, APF, GPF, and TSH were further illustrated through smooth curve fittings and generalized additive models **(Fig 2)**. Upon sex stratification, the relationships were separately depicted **(Fig 2D–2F)**. In addition to the previously noted significant positive correlation in females, the relationship between APF and TSH in males exhibited a U-shaped curve, with the inflection point at APF = 32.6%.

## Discussions

Our study, analyzing NHANES 2007–2012 data for adults aged 20 years and above, demonstrated a significant positive association between TPF and TSH in the fully adjusted model. However, the positive correlations of APF and GPF with TSH were not statistically significant. In sex-stratified analyses, TPF, APF, and GPF maintained significant positive associations with TSH in females across all three models. In contrast, in males, these relationships were negative, although not statistically significant. Notably, a U-shaped non-linear relationship with an inflection point at 36.2% was identified between APF and TSH in males.

Previous studies investigating the relationship between crude measures of adiposity, such as BMI, and TSH have yielded varying results. For instance, two prospective studies, with 937 [12] and 1,100 [13] participants respectively, found no clear relationship between BMI and TSH. In contrast, a study of 5,009 participants [8] reported a significant positive association, while another study with 1,564 participants [14] identified a positive but statistically non-significant association. Meanwhile, a third study of 2,524 participants [15] concluded that there was no clear relationship between these variables.

The relationship between TSH and obesity, as measured by DXA, also yields diverse conclusions. Adamska [16] discovered a negative association between serum TSH and visceral adipose tissue (VAT) in men with a BMI less than 25 kg/m2, while this relationship disappeared in men with BMI greater than 25 kg/m2 and the women’s group. However, Witte [17] found no significant association between TSH and VAT. Examining potential sex differences, Friedrich [18], consistent with our study’s findings, identified a positive correlation between waist-to-hip ratio, BMI, waist circumference, hip circumference, and TSH concentration in women, which was not observed in men.

The divergence in previous study results may stem from variations in the measurement of obesity. Body composition assessed by DXA is considered more accurate and informative compared to parameters like BMI. This precision is particularly relevant given the close association between visceral fat and adipocytokine production, insulin sensitivity, and the heightened risk of diseases such as diabetes and hypertension [19]. Beyond the method of measuring obesity, factors like study design, sample size, the method of TSH measurement, thyroid function of the subjects, and overall obesity of the subjects can contribute to the observed variation in results. Additionally, underlying ethnic differences across study cohorts could be another driver of heterogeneity in these findings. For instance, in our study, we accounted for participants’ racial backgrounds, as shown in Table 1, which includes Hispanic, non-Hispanic White, non-Hispanic Black, and other racial groups. To address potential differences among these groups, we adjusted for race as a covariate in our regression analyses. Despite the diverse outcomes in the relationship between TSH and obesity across studies, it is evident that thyroid function and obesity share a distinct yet intricate association [20].

The mechanisms underlying the positive correlation between TSH and body fat, as demonstrated in our study, are intricate. Existing research remains uncertain about whether elevated TSH is a cause or a consequence of obesity development. TSH, as a component of the hypothalamic-pituitary-thyroid axis (HPT axis), plays a vital role in metabolism, and its impact on energy metabolism has been extensively studied. As an upstream signal for thyroid hormones, TSH influences thyroid hormones, which regulate 60% of daily basic energy expenditure [21], thereby exerting an influence on energy metabolism. Furthermore, TSH can act directly by binding to its receptors, the G-protein-coupled TSH receptor (TSHR), present in human white adipocytes (WAT) and brown adipocytes (BAT) [22]. The TSH/TSHR interaction is believed to directly impact the normal function, adipogenesis, and the balance between lipolysis and lipogenesis in adipose tissue [23]. Additionally, studies have shown that in individuals with normal thyroid function, elevated TSH levels are positively correlated with triglyceride levels and markers of insulin resistance [24], suggesting that TSH also affects fat deposition by influencing glucolipid metabolism.

Leptin is considered a primary factor contributing to elevated TSH levels in obese patients [25]. Its pivotal metabolic functions involve activating the synthesis of TSH and thyroid hormones by directly or indirectly inducing TRH expression, regulating thermogenesis through the neuronal pathway, promoting satiety and appetite suppression, and enhancing metabolism by activating thyroxine deiodinase (DIO) expression and increasing the conversion of thyroxine (T4) to triiodothyronine (T3) [26]. Additionally, leptin plays roles in stimulating bone formation and regulating immune cells. Leptin secretion by adipocytes is proportional to the mass and nutritional status of adipose tissue, with higher secretion observed in subcutaneous adipose tissues compared to visceral adipose tissues [27]. In individuals with obesity, the surplus energy, elevated leptin secretion, and down-regulation of leptin receptors lead to leptin resistance [28]. This resistance results in hyperleptinemia and, consequently, an increase in TSH levels.

Lipotoxicity, characterized by the deposition of lipids in tissues other than adipose tissues in obese individuals with hypertriglyceridemia, could also contribute to the observed results [29]. Furthermore, adipose tissue secretes pro-inflammatory adipokines such as TNF-α and IL-1, which can reduce thyroid hormones secretion by blocking the expression of sodium-iodine transporter protein mRNA in thyroid cells. This, in turn, leads to a compensatory increase in TSH levels [30]. Recent studies propose that Fibroblast Growth Factor 21 (FGF21), produced by the liver and stimulating processes like lipolysis, may serve as another link between adipose tissue and the HPT axis [31]. In summary, TSH affects energy expenditure and changes in adipocytes through pathways involving thyroid hormones. As BMI increases, obesity can also influence the HPT axis through factors such as leptin, lipotoxicity, and inflammatory adipokines. However, the causality of the relationship between the HPT axis and obesity remains unclear.

Our study revealed significant sex differences with limited clarification, consistent with findings in some of the previously mentioned studies. One plausible explanation is that the thyroid appears to be more responsive to the physiological changes associated with increased fat in females. A notable characteristic of thyroid disorders is their higher prevalence in women compared to men [32].This disparity may be linked to estrogen’s regulatory role in the immune system [33] and the inactivation of the X chromosome [34]. This thyroid instability could render females more susceptible to hypothyroidism, and consequently, elevated TSH may be more pronounced in the presence of fat accumulation [35]. Furthermore, sex hormones play a role, with estrogen positively impacting leptin secretion in female adipose tissue, while testosterone and dihydrotestosterone exert the opposite effect. These hormonal influences may contribute to sex differences in TSH. Although the physiological significance of higher leptin levels in women remains unclear, it is noteworthy that women exhibit higher TSH levels than men in BMI-matched normal populations.

Lastly, our study identified an inflection point between TSH and APF in male subjects. While not achieving statistical significance, the underlying reason for this phenomenon warrants further investigation. This observation may suggest that the impact of fat on TSH varies by region. Recent research has explored the relationship between the visceral adiposity index (VAI), a relatively new and effective method for assessing central visceral obesity distribution, and adipocyte function with TSH. In this investigation, VAI exhibited a significant positive correlation with TSH levels. Nonetheless, this unusual U-shaped relationship demands additional research for a more comprehensive understanding.

## Conclusion

This study revealed a statistically significant positive association between TSH and TPF in adults aged 20 years and above. Similarly, in females, significant positive correlations were observed between TSH and adiposity measures, including TPF, APF, and GPF. While the opposite negative associations in males did not reach statistical significance, a U-shaped non-linear relationship was identified between TSH and APF, with an inflection point at 32.6%.

## Strength and limitations

The main strengths of our study lie in the utilization of a large, nationally representative population of U.S. adults, ensuring comprehensive data on body fat composition (TPF, APF, GPF) and TSH from NHANES. Additionally, the study employed DXA for measuring fat content, enhancing the robustness and suggestiveness of the results. The considerable sample size allowed for stratification by sex and other factors, revealing sex differences in the relationship between TSH and obesity. However, the study has limitations, primarily due to its cross-sectional nature, which precludes the establishment of a causal relationship between TSH and obesity. Long-term follow-up studies would be essential to substantiate causality. Furthermore, the sex differences observed in our study, as well as the identified U-shaped curves in male subjects, lack clear explanations and necessitate further investigation.
